# Biological Rhythms, Chrono-Nutrition, and Gut Microbiota: Epigenomics Insights for Precision Nutrition and Metabolic Health

**DOI:** 10.3390/biom14050559

**Published:** 2024-05-06

**Authors:** Nathalia Caroline de Oliveira Melo, Amanda Cuevas-Sierra, Vitória Felício Souto, J. Alfredo Martínez

**Affiliations:** 1 Department of Nutrition at the Federal University of Pernambuco, Recife 50670-901, PE, Brazil; nathalia.melo@ufpe.br (N.C.d.O.M.); vitoria.felicio@ufpe.br (V.F.S.); 2 Precision Nutrition Program, Research Institute on Food and Health Sciences IMDEA Food, CSIC-UAM, 28049 Madrid, Spain; jalfredo.martinez@imdea.org; 3 Centro de Investigación Biomédica en Red Fisiopatología de la Obesidad y la Nutrición (CIBEROBN), Instituto de Salud Carlos III, 28029 Madrid, Spain; 4 Centre of Medicine and Endocrinology, University of Valladolid, 47002 Valladolid, Spain

**Keywords:** body clock, circadian rhythm, genomic, environmental adaptation, microbiota

## Abstract

Circadian rhythms integrate a finely tuned network of biological processes recurring every 24 h, intricately coordinating the machinery of all cells. This self-regulating system plays a pivotal role in synchronizing physiological and behavioral responses, ensuring an adaptive metabolism within the environmental milieu, including dietary and physical activity habits. The systemic integration of circadian homeostasis involves a balance of biological rhythms, each synchronically linked to the central circadian clock. Central to this orchestration is the temporal dimension of nutrient and food intake, an aspect closely interwoven with the neuroendocrine circuit, gut physiology, and resident microbiota. Indeed, the timing of meals exerts a profound influence on cell cycle regulation through genomic and epigenetic processes, particularly those involving gene expression, DNA methylation and repair, and non-coding RNA activity. These (epi)genomic interactions involve a dynamic interface between circadian rhythms, nutrition, and the gut microbiota, shaping the metabolic and immune landscape of the host. This research endeavors to illustrate the intricate (epi)genetic interplay that modulates the synchronization of circadian rhythms, nutritional signaling, and the gut microbiota, unravelling the repercussions on metabolic health while suggesting the potential benefits of feed circadian realignment as a non-invasive therapeutic strategy for systemic metabolic modulation via gut microbiota. This exploration delves into the interconnections that underscore the significance of temporal eating patterns, offering insights regarding circadian rhythms, gut microbiota, and chrono-nutrition interactions with (epi)genomic phenomena, thereby influencing diverse aspects of metabolic, well-being, and quality of life outcomes.

## 1. Introduction

Epigenomics characterizes a set of reactions and processes that regulate changes (activation or suppression) in the functions of genes without altering the sequences of the nitrogen bases (adenine, guanine, cytosine, and thymine) of the DNA (deoxyribonucleic acid) molecule [1,2], which leads to distinctions between the phenotypical expression of cell groups that have the same genome background [3].

Epigenetic regulation occurs mainly through the action of compounds that bind to DNA during the demethylation/methylation reaction or to histone proteins during the acetylation process or other radicals [2]. These phenomena are mediated by enzymes (such as DNA methyltransferase, histone acetyltransferase, and histone deacetylase), which favor or compromise these connections. The product of these bonds is a more compact chromatin (heterochromatin), which impairs DNA transcription, or a looser chromatin (euchromatin), which favors the binding of transcription factors and the regulation of cellular pathways [4].

In addition to epigenetic variations associated with DNA methylation and the acetylation of histone proteins, the suppression or promotion of DNA translation into protein can be influenced by post-translational reactions of histones as in the ubiquitination, phosphorylation, and biotinilation stages [4], and/or by the action of ncRNA (ribonucleic acid not coding molecules) [5] of the micro type (miRNA), which regulates the gene silencing/activation during the transcription process and/or regulates the translational level of protein-coding genes or long type (lncRNA), which acts in chromatin remodeling as a transcriptional regulator and/or as post-transcriptional regulators [6].

The technological advancement and generation of high-resolution genetic sequencing allowed the identification of numerous molecules and reactions associated with epigenetic interactions [7]. A critical gap remains concerning the interactions between environmental, physiological, and biological factors impacting chromatin dynamics across the lifespan, making epigenomics a compelling subject for ongoing scientific research [8,9,10].

Epigenetic modulation becomes even more complex when considering the impact of chrono-nutrition, gut microbiota dynamics, and biological rhythms. Indeed, emerging evidence suggests that the timing of nutrient/food intake, influenced by the circadian clock, may modulate epigenetic mechanisms [11]. Additionally, the gut microbiota, closely linked to circadian rhythms, plays a pivotal role in metabolizing nutrients and producing bioactive compounds that can influence epigenetic homeostasis [12,13]. As these multifaceted elements converge, an in-depth exploration of putative interactions will uncover novel insights concerning the influence of lifestyle factors in shaping the epigenome and, consequently, impacting the overall metabolic state and health.

This review aims to elucidate the integrated epigenetic network orchestrating the circadian control of biological rhythms, nutritional cues, and gut microbiota dynamics, thereby unravelling the consequential implications for personalized and precision health.

## 2. Biological Rhythms and Epigenetic Regulation

For thousands of years, human beings preferentially carried out life activities during exposure to light and rested at night. The repetition of various biological processes at the central and peripheral levels characterizes the circadian rhythm (CR), which corresponds to a variety of processes that persist daily in constant conditions [14], such as physiological, metabolic, and behavioral processes that occur 24 h a day [15].

Biological rhythmicity is observed both in mammals and in primitive beings, such as bacteria [16] and consists of an integrated, regulated, and self-sustaining network of trends displayed in all cells of the body, with an important role in synchronizing body physiology and behavior [17] from the molecular to the environmental level [18].

The rhythmic expression of these biological systems occurs through the action of the so-called “clock genes” expressed in tissues such as the liver, heart, brain, and gut [19], which send signals to the central circadian regulator based on the suprachiasmatic nucleus (SCN) located in the anterior region of the hypothalamus, responsible for the circadian integration of information coming from peripheral tissues, which is also directly influenced by light or dark stimuli and regulatory neuroendocrine signals [18].

These internal clocks exhibit genetically determined, endogenous temporal patterns, even in the absence of external cues, through transcriptional-translational feedback mechanisms [20], and the primary genes most studied are the following: CLOCK (circadian locomotor output cycles kaput), BMAL1 (brain and muscle aryl hydrocarbon nuclear receptor translocator like 1), Per (Period), and CRY (Cryptochrome) [19], which, through heterodimerization and nuclear processes, modulate gene transcription in the cell nucleus after the translocation of CRY and Per in the cytoplasm, forming a feedback complex of transcriptional activity signal [21].

**Figure 1:**
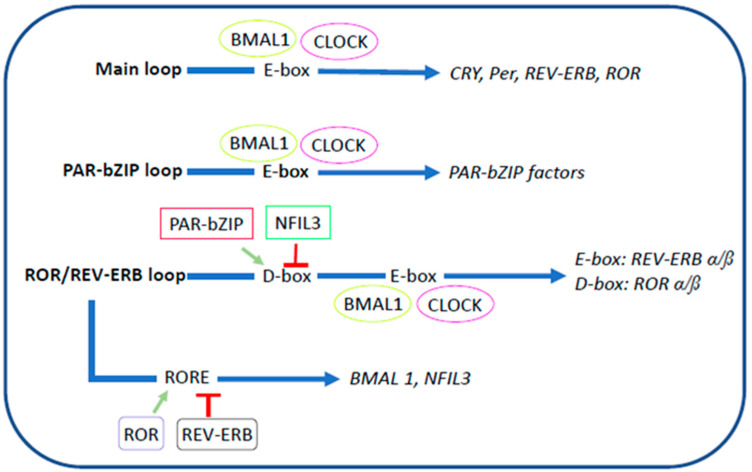
Molecular transcription cascade of clock genes. CLOCK: Circadian locomotor output cycles kaput, BMAL1: brain and muscle aryl hydrocarbon nuclear receptor translocator like 1, Per: period, CRY: cryptochrome, ROR: retinoic acid related orphan receptor, PAR-bZIP: proline and acidic amino acid-rich basic leucine zipper protein, NILF3: nuclear factor interleukin 3 regulated, DBP: D-box binding protein, TEF: thyrotroph embryonic factor, HLF: hepatic leukemia factor. Adapted from Schurhoff and Toborek, 2023 [21].

The molecular cascade involving clock genes occurs in the nucleus and is composed of three interdependent phases involving the transcription of orchestrated components: (1) in the main loop, BMAL1 and CLOCK heterodimerize and bind to E-box regions to induce the transcription of CRY, Per, REV-ERB, and ROR, which, after their accumulation, heterodimerize, inhibiting their own transcription; (2) in the PAR-bZIP loop, BMAL1 and CLOCK bind to the E-box regions to modulate the transcription of the factors associated with PAR-bZIP (such as DBP, TEF, and HLF); and (3) in the ROR/REV-ERB loop, PAR-bZIP binds to the D-box region, activating the E-box region, where BMAL1 and CLOCK promote the transcription of REV-ERB and ROR. Then, ROR stimulates the transcription of RORE to regulate and inhibit the transcription of BMAL1 and NFIL3 (Figure 1) [21].

The circadian characteristics of each person are related to their tendency to perform activities (e.g., work or sport) at certain moments of the 24 h cycle, which makes it possible to classify individuals into different chronotypes [22]. Individuals may be morning type, preferring to carry out their activities from dawn to midday; afternoon type, having a maximum productivity from the afternoon to dusk; or nocturnal type, working optimally during the night and early morning [23], which enables their peripheral clocks to respond differently to environmental stimuli [22].

The SCN integrates the information captured by the sensory systems (vision, smell, hearing, touch, and taste), as well as receiving and sending information to peripheral tissues (liver, pancreas, heart, and gut) through neuroendocrine and non-humoral signals, acting at a central level as the main circadian regulator of all biological systems [24]. Circadian regulation is resistant and “adaptable” to short-term environmental changes. These changes, when they are chronic, result in a cascade of endogenous shifts at different levels of regulation of circadian cues, central and peripheral, which interact with each other [25].

In human and experimental models, different environmental and physiological stimuli can influence the regulation of CR through epigenetic adjustments, especially those related to the promotion of DNA methylation and histone acetylation reactions (Table 1), and these adjustments are reflected by the expression of distinct phenotypes.

## 3. Chrono-Nutrition and Epigenetic Interactions

**Figure 2:**
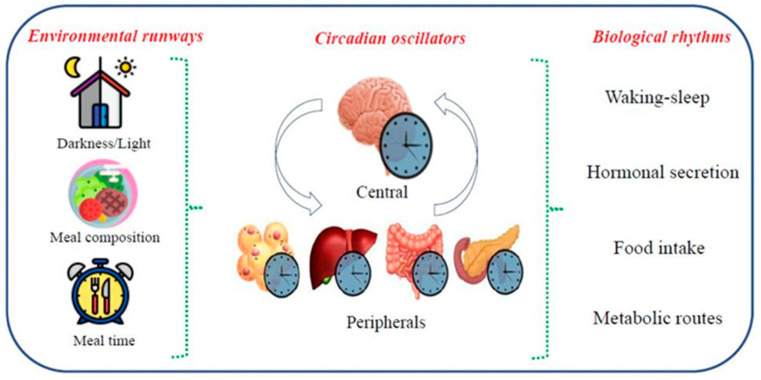
Chrono-nutrition and regulation of the dietary circadian rhythm. The presence or absence of light, meal composition, and/or time variation between the first and last meal of the day act as environmental cues captured by the central circadian regulator (SCN) and peripheral circadian regulators (white adipose tissue, liver, intestine, and pancreas) that interact with each other and are involved in the regulation of biological rhythms associated with food intake (hormonal regulation of hunger-satiety; regulation of the digestive and absorptive process; metabolism and use of nutrients and their serum concentrations). BioRender.com.

Among the environmental variables, the integration between light/dark cycle exposure and nutrition, not only by the composition of the diet, but mainly by the time the meal is performed, has emerged as an important modulator of CR in different systems [39]. Nutrition corresponds to a set of involuntary and unconscious biological processes by which organisms digest, absorb, metabolize, and utilize nutrients for their survival [40]. In the same way, biological clocks are regulated by a set of cyclical physiological and metabolic factors over the 24 h that interact with the timing, frequency, and composition of meals, characterizing the circadian food intake distribution (Figure 2), giving rise to what is known as chrono-nutrition [41,42].

In addition, the occurrence of macronutrients (carbohydrate, lipids, and protein) in the meal and the time they are consumed act as a key point to align the metabolic repercussions of food intake with the action of an integrated complex of neural and peripheral structures associated with food and called FEO (food-entrainable oscillator) [43,44,45]. The FEO characterizes an independent and self-sufficient system directly influenced by food and located outside the SCN [46]. Although the hypothetical mechanisms of action are not completely elucidated in the literature, the putative functionality is linked to predicting the moment of feeding, which leads to the preparation of the tissues of the gastrointestinal tract, promoting the quotidian synchronization between food or beverage intake with the digestive process and absorptive and nutrient utilization over 24 h [47].

Chrono-nutrition is very sensitive to environmental signals, which applies to lifestyle changes, such as skipping breakfast or eating more carbohydrates at night, which impact the circadian dysregulation (chrono-disruption) of food intake by dragging (delaying or advancing) the action of the genes of the circadian machinery in distinct tissues, in the central (SCN) or at the peripheric level [48], as in the liver or pancreas. This circadian flexibility has been associated with the development of different metabolic conditions, such as insulin resistance. However, on the other hand, it can be used as a preventive or therapeutic agent for the treatment of chronic non-communicable diseases [11,49] since the time of food intake (daytime versus nighttime) and the availability of substrates from the diet [such as Acetyl CoA, NAD (nicotinamide adenine dinucleotide), and SAM (s-adenosyl methionine)] act as precursors/intermediates (cofactors/substrates) of DNA methylation reactions and histone acetylation or deacetylation, acting as a modulator of chromatin characteristics [50].

The day-to-day realignment of endogenous genes [specifically BMAL1, CLOCK, and NR1D1(nuclear receptor subfamily 1 group D member 1)], via the regulation of DNA methylation reactions observed at different CpG sites, can be induced by the adoption of the mediterranean diet and favors the regulation of energy homeostasis and weight loss [51,52]. Likewise, in recent years, nutritional strategies associated with daily caloric control [53] and the consumption of foods with functional properties, such as extra virgin olive oil, were directly related to the regulation of DNA methylation reactions, the prevention or treatment of obesity, and associated comorbidities.

Changes to dietary patterns and mealtimes impacts DNA methylation [54] and miRNA pathways, as observed by Quintanilha et al. [55] and peripheral blood, tissues, and cellular samples, where, within dietary patterns, the manipulation of the diet through the consumption of isolated nutrients or bioactive compounds (for example, resveratrol, curcumin, and polyunsaturated fatty acid) influences gene expression thanks to the epigenetic pathway associated with miRNAs (Table 2), which are usually involved in the silencing of post-transcriptional genes, inducing mRNA degradation or repression by binding to a targeted messenger RNA and helping to prevent the development of chronic diseases, such as type 2 diabetes and cardiovascular diseases.

In parallel, there is a growing adoption of intermittent fasting (IF). IF is already known in the context of the religious practices of Islam (Ramadan fasting), but has been gaining popularity for characterizing a nutritional strategy for weight/adiposity loss and metabolic control associated with eating during a daily food window followed by fasting [56], resulting in: (I) weight reduction/maintenance; (II) metabolic flexibility (switching from using glucose as an energy source to using fatty acids and ketone bodies, leading to a reduction in adipose tissue); (III) mitochondrial biogenesis; (IV) DNA repair; (V) autophagy of damaged/diseased cells; and (VI) resistance to metabolic, oxidative, and nutritional stress [57], potently involving epigenetic markers.

Among the IF protocols, time-restricted feeding (TRF) stands out. It “allows” the consumption of food in specific “time windows”, with windows of 8 to 20 h of daily fasting. The ratio more commonly used is the 16:8 sub-protocol, in which the individual spends 16 h fasting and has 8 h in which they are free to access food [58]. In 2020, Templeman et al. [59] suggested that reducing caloric intake at night and fasting for longer night periods (fasting in the inactive phase) is related not only to a decrease in systemic metabolic inflammation but also to quotidian realignment, improving the individual’s health and quality of life in a general way. Accordingly, it was found that TRF allows the control of nutritional cues that interact with peripheral clocks, helping them to maintain the optimized day-to-day rhythms, in addition to being a low-cost strategy for the prevention and/or reversal of metabolic imbalances in rodents and humans [60]. Intriguingly, circadian feeding behavior and metabolic processes remain preserved in animals that maintained the TRF cycle in the presence of SCN damage, highlighting the non-photonic and possibly independent influence that TRF has on peripheral oscillations and circadian regulation [61].

Currently, TRF can confer health benefits. One of the mechanisms proposed is the putative ability to modulate epigenetic pathways, especially those related to the inhibition of miRNAs 122, 143, and 222, which are considered endocrine biomarkers and targets [62] in blood samples of humans and animals [63]. These miRNAs act as epigenetic intercellular communicators, favoring adipogenesis, for example, being related to metabolic disorders such as obesity [62].

In the study by Saini et al. [64] on the overweight elderly, 14 miRNAs were differentially expressed when comparing the pre- and post-TRF period. Specifically in the post-TRF period, targets of regulated miRNAs suggested the increased expression of (1) PTEN (classical DNA repair-related gene) [65], TSC1 (tumor growth suppressor) [66] and ULK1/2 (autophagy activating protein kinase) [67], which are related to cell repair and survival; (2) Ras protein, which regulates the mitogen-activated protein kinase pathway and phosphoinositide-3 kinase, which are involved in the control of cell growth and survival. Interestingly, these pathways are inhibited in tumor cells, suggesting that the potential beneficial effect of TRF on the cancer population should be evaluated in future studies; (3) mTOR (cell growth and protein synthesis); (4) insulin sensitivity (glucose uptake); and (5) the endophagic process (cell homeostasis and survival) [64]. Together, these findings suggest that TRF can inhibit uncontrolled cell growth pathways and activate survival pathways such as autophagy and cell repair, promoting good health. This relationship demonstrates that health is directly related not only to what you eat, but also to the time window in which food is consumed, mediated by epigenetic involvement.

Interestingly, all these synchronization/desynchronization interactions of endogenous biological clocks in which food intake time windows modulate epigenetic routes that also affect gut physiology and the health of gut microbiota (GM), which corresponds to a set of microorganisms (bacteria, viruses, fungi, protozoa, and archaea) that inhabit both the small intestine and colon, which are involved not only in the metabolic and immunological functions of their host [68], but also critically in circadian regulation through multidirectional interaction between its metabolites, diet, and clock genes [18].

## 4. Gut Microbiota and Epigenetic Modulation

Regardless of the time of meal intake, the intestinal lumen comprises the last and main layer of contact between the gastrointestinal tract and nutrients in their absorptive form [69]. The small intestine is composed of different types of cells, where enterocytes stand out (absorptive cells present along the epithelium); goblet cells (responsible for the production of mucins, proteins capable of providing not only a protective barrier and molecular exchange between the environment and the intestinal epithelium); and Paneth cells (secretors of antimicrobial products when the epithelium detects external microbial fragments), being a key tissue of metabolic homeostasis [68]; and defense functions [69].

In this context, the gut houses 70–80% of immune cells and represents an important lymphoid tissue rich in Peyer’s patches, which secrete CD4/CD8 T lymphocytes, plasma cells (secretors of immunoglobulin A), macrophages, and dendritic cells. Above Peyer’s patches are the M cells that perform endocytosis, in addition to the mesenteric lymph nodes and an extensive surface with diffuse lymphocytes, which reflects the interaction between the gut and the immune system [70], making it essential in the daily fight against pathological agents and in the mediation of the low-grade chronic inflammatory process.

**Figure 3:**
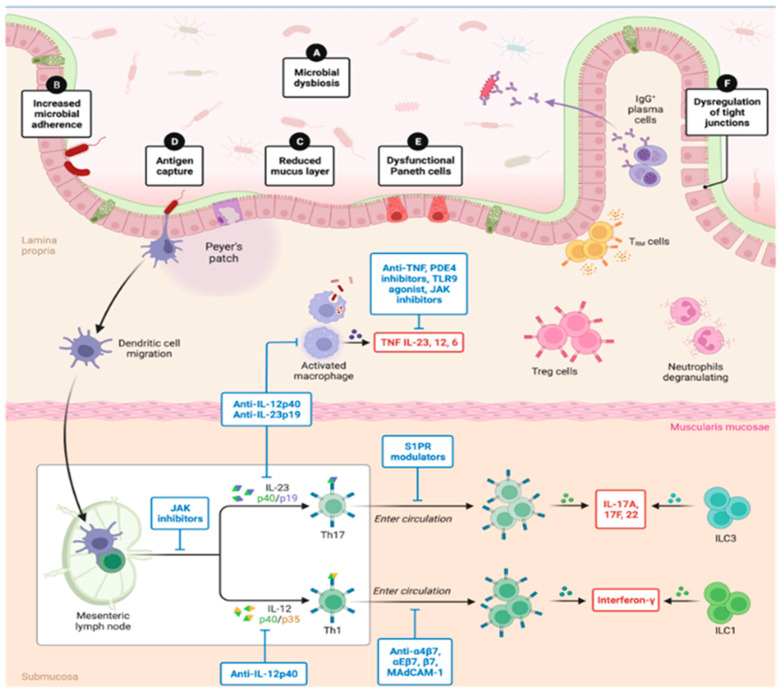
Interactions between the gut epithelial barrier and the anti- and pro-inflammatory cascade. TNF: Tumor necrosis factor. PDE4: Phosphodiesterase 4. TLR9: Toll-like receptor 9. JAK: Janus kinase. IL: Interleukine. Treg cells: Regulatory T cells. Th17: T-helper type 17 cell. Th1: T-helper type 1 cell. S1PR: G-protein-coupled S1P receptor. BioRender.com (2023) and adapted from Chang [74].

Although the inflammatory process associated with poor diet and obesity occurs systemically, intestinal homeostasis is a key condition in the regulation of the state of organic stress [71,72]. The epithelial layer of the gut protects against the passage of intestinal microbes, food antigens, and toxins that reach the lumen [72]. However, susceptibility to intrinsic and extrinsic factors (such as genetic predisposition, dietary pattern, the use of antibiotics, or interruption to the circadian rhythm) can lead to the translocation of distinct components, such as lipopolysaccharide, to the lamina propria, instigating the onset of anti- and pro-inflammatory mechanisms correlated with the pathogenesis of the chronic inflammatory state [73] (Figure 3).

Concomitantly, the gut its closely associated with the FEO complex, and its circadian functionality is linked to the action of other regulatory clock genes, such as CLOCK and Per1 (Period 1), which regulate gut motility [46], and must be regular and optimized for the arrival of nutrients in the lumen, a key fact that drives the secretion and action of gastrointestinal hormones that regulate the circadian feeding rhythm [75].

In general, all cell membrane transporters found in the gut are regulated by the presence/absence of nutrients in the intestinal lumen, which also triggers the regulation of hormone secretion rhythms [76]. Thus, interrupted/altered feeding rhythms, such as reduced food intake during the day and increased food intake during the night result in altered postprandial responses and the impairment of hormone secretions involved in the digestive process and metabolic control, such as insulin [75].

When in contact with nutrients, the intestinal mucosa is exposed to a large amount of bacterial and invading antigens, usually from food, and the function of the intestinal mucosal barrier is to provide an immune defense [68]. This immune function of the gut is dependent on the interaction between the integrity of the gut barrier, the effectiveness of the immune system and the GM (set of microorganisms, especially bacterial groups, that colonize the small intestine and colon and have a symbiotic relationship with the host, with an important influence on metabolism regulation) [77,78]. On the other hand, the relationship between microbial metabolism and circadian regulation has been of scientific interest for the elucidation of epigenetic pathways associated with GM and may impact on precision nutrition management and health [79].

In this context, it is known that GM influences the physiology of hosts through three potential epigenetic mechanisms: (1) the availability of chemical donors for DNA methylation or histone protein modifications; (2) the regulation of the expression and/or activity of enzymes involved in epigenetic mechanisms; or (3) the activation of processes intrinsic to host cells that direct epigenetic pathways [13].

The GM can synthesize several biological components. Among them, methyl and acetyl groups have been identified as the substrates necessary for DNA methylation reactions and the acetylation of histone proteins, respectively [80]. The donation of these groups occurs thanks to the power of the GM to synthesize epigenetic substrates (cofactors or enzyme regulators involved in epigenetic reactions), such as folate and B vitamins synthesized by *Bifidobacterium* and *Lactobacillus*, mainly, which are associated with the donation of methyl groups [81].

Similarly, commensal microbes ferment complex carbohydrates and fibers, producing short-chain fatty acids such as butyrate, which represent an important group of epigenetically relevant molecules associated with the inhibition of histone deacetylases, especially histone deacetylase class I (HDAC3—histone deacetylase type 3), which is highly expressed in the gut epithelium and sensitive to microbial signals, associated with homeostasis of the Paneth cells and the protective function of the intestinal barrier [13], resulting in changes in chromatin configuration [82]. Concomitantly with enzyme inhibition, short-chain fatty acids also contribute to the increased cellular levels of acetyl coenzyme A by regulating the action of dioxygenases involved in DNA methylation [83].

The GM largely impacts DNA methylation in various cell types and tissues in the health-disease axis [84,85,86]. Through transcriptomics and DNA methylation analysis in germ-free versus colonized mice it was possible to demonstrate that the microbiota induced DNA hypomethylation and increased the expression of anti-bacterial and anti-inflammatory genes, promoting the metabolic homeostasis of these animals [87].

Acetylation and histone methylation have been the most studied pathways in the microbial regulation scenario. These modifications are balanced by the activity of opposite classes of epigenetic enzymes (histone deacetylases versus histone methyltransferases, for example). It is important to emphasize, however, that new studies are necessary to gain a clearer understanding of the interactions between these enzymes and the reactions of chromatin and methylation, since they are involved in the relationships between the GM and the host [88].

Noteworthy, Krautkramer et al. [89] reported that gut microbiota alters the acetylation and methylation of histones H3 and H4 in various tissues (colon, liver, and adipose tissue) in a diet-dependent manner and the supplementation with short-chain fatty acids in rats was able to partially restore histone changes, suggesting a potential therapeutic target based on epigenetic screening.

In addition to microbial interaction with specific epigenetic marks, GM can indirectly influence epigenetic mechanisms by activating immune cells, such as macrophages and dendritic cells, which are essential for the transcription cascade of genes involved in interferon and T cell signaling, which is another mechanistic pathway that relates the GM to the immune response [90]. Likewise, the GM can induce the expression of IncRNAs in the thymus and spleen [91], which suggests another epigenetic pathway where GM acts out of the gut as a modulator of the immune system, highlighting the need for further studies in the area [92,93].

## 5. Concluding Remarks

The feeding schedule and quotidian metabolic function over 24 h has a daily impact on the synchronization of physiological processes regulated by clock genes throughout the body, specifically in the gastrointestinal tract and associated systems, such as GM. In turn, the interactions between the intestinal microorganisms and their host have nutritional, immunological, and metabolic effects on a day-to-day basis associated with health maintenance and the installation and/or control of chronic non-communicable diseases by the modulation of the inflammatory state and other regulatory pathways, highlighting and emphasizing the importance of multidirectional interactions between nutrients, the circadian system, and GM interfaces.

Heterochromatin and multi-omics studies concerning the identification of changes in the configuration of the DNA, the genetic sequencing of GM, and the expression of clock genes in different tissues have shown that this interaction (nutrient and mealtime/biological rhythm/GM) is multidirectional and modulated by epigenetic mechanisms that occur concurrently at different cellular levels and organic systems, being an emerging and complex area for renewed scientific research for precision nutrition.

Although many studies in humans and experimental models provide data on circadian synchronization/desynchronization, gut microbial composition, epigenetic pathways, and the associated repercussions on the health-disease process, future perspectives should stimulate an aim to elucidate mechanistic pathways and network involving epigenetic interactions, not only in the gut microbiota-host relationship, but also within the microbial community itself. Furthermore, potential therapeutic targets that can be modulated by the synchronization of circadian rhythms and gut microbiota will involve putative changes in lifestyle, particularly related to daytime meals and the adoption of healthy eating patterns, based on epigenetic signatures and modulation for precision health maintenance.

## Figures and Tables

**Table 1:** Environmental and physiological factors that modulate circadian regulation through epigenetic interaction.

Environmental or Physiological Factor	Epigenetic Interaction	Circadian Regulation	Reference
Reduction of the light/darkness cycle	DNA Methylation *	Disturbances in the transcription cascade of clock genes in the SCN	[26]
Exposure to light in inactive phase	Histone Acetylation *	↑ Per (1 and 2)/CRY1	[27]
Night Workers	DNA Methylation *	↓ do CLOCK ↑ do CRY2	[28]
Senescence	DNA Methylation *	↑ of CpG sites **	[29,30]
Cortisol levels and GR	Regulate different sets of transcripts in a spatial domain of chromatin *	Regulates transcriptional activity of Per and CRY	[31,32]
Physical activity	DNA Methylation *	Regulator of CRY, BMAL1, and PPAR-δ	[33,34]
	Length of telomeres *		[35,36,37]

GR: Glucocorticoid receptors. * The mechanism of epigenetic interaction was through the analysis of different tissues, and total blood was used in most cases. ** CpG sites: Characterize a region of non-methylated DNA and act as “sensors” of DNA methylation fraction in cells [38]. ↑: Increase. ↓: Decrease.

**Table 2:** Nutrients and epigenetic modulation of metabolic health by miRNA mechanisms.

Nutrient	Epigenetic Mechanism by miRNA Regulation	Metabolic Health
Resveratrol	↑ miRNA 663, miRNA Let7A ↓ miRNA 155, miRNA 21	↓ Inflammatory state
Saturated fatty acids in excess (high fat diet)	↑ miRNA 29a	Insulin resistance and type 2 diabetes
Polyunsaturated fatty acids	↑ miRNA 130b ↓ miRNA-146a, miRNA-146b, miRNA-21, miRNA-125a, miRNA-155 and miRNA 221	↓ Inflammatory state ↓ C-reactive Protein
Curcumin	↓ miRNA 155	↓ Inflammatory state
	↑ miRNA-181b, miRNA-146b-5p	↓ Inflammatory state and protect against the tumoral process
Vitamin D	↑ miRNA 125b, miRNA 100	Tumor suppression activity

miRNA: micro–Ribonucleic Acid. ↑: Increase. ↓: Decrease. Adapted by Quintanilha et al. [55].

## Abbreviations

Acetyl CoA: Acetyl coenzyme A. BMAL1: Brain and muscle aryl hydrocarbon nuclear receptor translocator like 1. CD4/8: Cluster of differentiation 4 or 8 of monoclonals cells. CLOCK: Circadian locomotor output cycles kaput. CpG sites: Regions of DNA where a cytosine nucleotide is followed by a guanine nucleotide in a linear sequence of bases along its 5′ -> 3′ direction. CR: Circadian rhythm. CRY: Cryptochrome. DBP: D-box binding protein. DNA: Deoxyribonucleic acid. FEO: Food-entrainable oscillator. GM: Gut microbiota. GR: Glucocorticoid receptors. HDAC3: Histone deacetylase type 3. HLF: Hepatic leukemia factor. IF: Intermittent fasting. IL: Interleukine. JAK: Janus kinase. lncRNA: Long-type of ribonucleic acid not coding. miRNA: Micro-ribonucleic acid. mRNA: Messenger RNA. mTOR: Mammalian target of rapamycin. NAD: Nicotinamide adenine dinucleotide. NcRNA: Ribonucleic acid not coding. NILF3: Nuclear factor interleukin 3 regulated. NR1D1: Nuclear receptor subfamily 1 group D member 1. PAR-bZIP: Proline and acidic amino acid-rich basic leucine zipper protein. PDE4: Phosphodiesterase 4. Per: Period. PPAR-δ: Peroxisome proliferator—activated receptor gamma. PTEN: Phosphatase and tensin homologue. REV-ERB: Nuclear receptor REV-ERB. RNA: Ribonucleic acid. ROR: Retinoic-acid-related orphan receptor. S1PR: G-protein-coupled S1P receptor. SAM: S-Adenosyl methionine. SCN: Suprachiasmatic nucleus. TEF: Thyrotroph embryonic factor. Th1: T-helper type 1 cell. Th17: T-helper type 17 cell. TLR9: Toll-Like receptor 9. TNF: Tumor necrosis factor. Treg cells: Regulatory T cells. TRF: Time-restricted feeding. TSC1: Tuberous sclerosis 1. ULK1: Unc-51-like autophagy activating kinase 1.
